# Solutions to common issues in the use of LAIs

**DOI:** 10.1017/S1092852925100552

**Published:** 2025-12-23

**Authors:** Stephen R. Saklad

**Affiliations:** College of Pharmacy, Division of Pharmacotherapy & Translational Science, The University of Texas at Austin, San Antonio, Texas, USA

**Keywords:** Long-acting injectable antipsychotics (LAIs), adherence, injection technique, oral overlap, missed doses, adverse effects, drug interactions, patient-centered care

## Abstract

Long-acting injectable (LAI) antipsychotics are highly effective tools for managing serious mental illness, yet their clinical utility is often compromised by logistical and pharmacological complexities. This review serves as a practical guide to optimizing LAI therapy by addressing common clinical hurdles. Maintaining a consistent injection schedule is essential to successful treatment. To improve adherence, clinicians should implement proactive reminder systems—such as phone calls or text messages—and involve family or caregivers in the care plan. When injections are delayed, management strategies must be tailored to the specific medication and the length of the “dosing window”. For example, aripiprazole monohydrate (Abilify Maintena) allows a ±7 day window, whereas paliperidone palmitate (Invega Sustenna) provides a +14 day window. If these windows are exceeded, catch-up protocols may involve administering the next dose as soon as possible, utilizing supplemental oral antipsychotics for a bridge period (e.g., 14 days for aripiprazole or 21 days for risperidone), or restarting initiation loading regimens entirely. Clinically significant drug interactions, such as the reduction of aripiprazole or risperidone levels by carbamazepine, can lead to symptom breakthrough. Conversely, CYP450 inhibitors like fluvoxamine or fluoxetine may increase antipsychotic concentrations, necessitating dose reductions. Adverse effects, including drug-induced Parkinsonism and akathisia, should be managed by reducing the LAI dose or switching to agents with lower risk profiles, such as aripiprazole-based products. For akathisia, short-term adjunctive treatments like vitamin B6 or mirtazapine may be utilized until dose adjustments reach steady state. Patient-centered care requires a collaborative approach to substance use, which can exacerbate symptoms or interfere with LAI effectiveness. Clinicians must also engage in nonjudgmental discussions when patients request a return to oral therapy, carefully considering the pharmacokinetic properties of the LAI to time the transition safely. Ultimately, a proactive management plan that addresses these clinical variables is essential for reducing relapse risk and improving long-term quality of life.

*Common situations encountered with the use of long-acting injectable (LAI) antipsychotics can be discouraging to clinicians. Some are more common and easily remembered, while others may be encountered less often. This article serves as a practical guide to addressing these issues.*

## Learning objectives

1. Identify common clinical challenges encountered when prescribing LAI antipsychotics.
2. Discuss strategies for addressing adherence, dosing, and missed injections with LAIs.
3. Evaluate approaches for managing adverse effects, drug interactions, and optimizing patient-centered outcomes.

## Storage and reconstitution requirements

LAIs have a range of storage requirements (Table 1). Some formulations, such as certain risperidone products, require refrigeration to maintain stability.^12^ ^,^^13^ In contrast, others, including paliperidone palmitate,^6^ ^–^^9^ aripiprazole,^3^ ^,^^4^ aripiprazole lauroxil,^1^ ^,^^2^ olanzapine,^5^ and different risperidone formulations,^10^ ^,^^11^ require storage at controlled room temperature (15 °C–30 °C). Specific products may have allowances for short-term storage outside their primary recommended range.^12^ ^,^^13^ Compliance with each product’s storage instructions is essential to maintain the drug’s stability and intended slow-release properties. These requirements are important not only for pharmacy and clinic storage but also for patients with planned travel that includes a prearranged injection site, as international travel may present additional concerns.^14^

**Table 1.** Storage and Reconstitution Requirements

Medication name (product)	Storage requirements	Reconstitution required
Aripiprazole Lauroxil (Aristada, Aristada Initio)^1^ ^,^^2^	Controlled room temperature	No
Aripiprazole Monohydrate (Abilify Maintena, Abilify Asimtufii)^3^ ^,^^4^	Controlled room temperature	No (Abilify Maintena also available as a vial that needs reconstitution)
Olanzapine Pamoate (Zyprexa Relprevv)^5^	Controlled room temperature	Yes
Paliperidone Palmitate (Invega Sustenna, Invega Trinza, Invega Hafyera, Erzofri)^6^ ^–^^9^	Controlled room temperature	No
Risperidone (Perseris, Risvan)^10^ ^,^^11^	Controlled room temperature	Yes
Risperidone (Uzedy)^12^	Refrigerator, 2 °C–8 °C (36 °F–46 °F); ≤90 d, 68 °F–77 °F (20 °C–25 °C); protect from light	No
Risperidone (Risperdal Consta)^13^	Refrigerator, 2 °C–8 °C (36 °F–46 °F); ≤7 d, ≤77 °F (25 °C); protect from light	Yes

Controlled room temperature is defined by the US Pharmacopeia (USP) to be thermostatically maintained between 20 °C and 25 °C (68 °F and 77 °F), with brief excursions from 15 °C to 30 °C (59 °F to 86 °F).

Some LAIs are formulated as lyophilized powders or liquids that require reconstitution prior to injection.^3^ ^,^^5^ ^,^^10^ ^,^^11^ ^,^^13^ The reconstitution process often involves product-specific techniques, such as vigorous shaking or the use of vial adapters, to ensure proper suspension. For example, olanzapine LAI necessitates a specific diluent and technique, including wearing gloves to prevent skin contact and eye protection from potential aerosolization.^5^ ^,^^15^ Conversely, many newer LAI formulations are available in prefilled syringes and do not require reconstitution.^1^ ^–^^4^ ^,^ ^6^ ^–^^9^ ^,^ ^12^ The type and volume of the diluent and the mixing protocol are critical and differ between products. Using multiple LAIs that need reconstitution in a single clinic can lead to errors; therefore, the clinician should review the specific product’s instructions before each use. Incorrectly reconstituted medications must be discarded.

## Injection site selection and technique

The appropriate injection site is an important aspect of LAI administration, as it can influence the rate of drug absorption.^16^ ^,^^17^ Injection volume and patient preference are key considerations. Most LAIs are given intramuscularly (IM) in either the deltoid or gluteal muscle. Some formulations with smaller volumes are specified for subcutaneous (SC) injection.

Since deltoid has significantly greater blood flow per volume of muscle than gluteus, initiating paliperidone palmitate in deltoid provides therapeutic drug concentrations sooner. Some LAIs, like olanzapine (Relprevv), are given by gluteal injection only. Higher doses of aripiprazole lauroxil and the paliperidone palmitate 6-month formulation require the gluteal muscle due to their large injection volume. Recording and rotating the injection site is advised (Table 2).

**Table 2.** Injection Sites, Technique, and Initiation Regimen

Medication name	Recommended injection site(s)	Injection technique	Oral overlap/initiation regimen
Risperidone LAI (Risperdal Consta)^13^	Deltoid or gluteal	IM	Yes (3 wk)
Paliperidone Palmitate (Invega Sustenna)^7^	Deltoid (initiation); deltoid or gluteal (maintenance)	IM	No/initiation doses
Paliperidone Palmitate (Invega Trinza)^8^	Deltoid or gluteal	IM	No (after ≥4 mo of Invega Sustenna)
Aripiprazole Monohydrate (Abilify Maintena)^3^	Deltoid or gluteal	IM	Yes (14 d) or two dose initiation
Aripiprazole Monohydrate (Abilify Asimtufii)^4^	Gluteal only	IM	No (if on Abilify Maintena currently) or yes (14 d) or two dose initiation
Aripiprazole Lauroxil (Aristada)^2^	Deltoid (441 mg); gluteal (≥662 mg)	IM (rapidly, do not aspirate)	Yes (21 d) or Aristada Initio
Aripiprazole Lauroxil (Aristada Initio)^1^	Gluteal only	IM	Used as single dose for initiation only
Olanzapine Pamoate (Zyprexa Relprevv)^5^	Gluteal only (deep)	IM	No
Paliperidone Palmitate (Invega Hafyera)^9^	Gluteal only	IM	No (after adequate treatment with Invega Sustenna or Invega Trinza)
Risperidone LAI (Perseris)^10^	Subcutaneous	SC	No (“double peak”)
Risperidone (Uzedy)^12^	Subcutaneous	SC	No (“double peak”)
Paliperidone Palmitate (Erzofri)^6^	Deltoid (first dose); deltoid or gluteal (maintenance)	IM	No (uses larger initiation dose)

Tolerability testing with the same oral medication for at least a few days is recommended to determine efficacy before using the LAI formulation. While aspiration to check for blood return before an IM injection is routinely recommended, it should be avoided with aripiprazole lauroxil products as this can clog the needle.^1^ ^,^^2^ If an aripiprazole lauroxil needle clogs, it should be replaced, and the injection administered rapidly at a different site. Recommendations for needle gauge and length depend on the specific LAI, the injection site, and patient factors like body weight.

## Oral overlap and initiation regimens

For several LAIs, an initial period of oral antipsychotic supplementation, or “oral overlap,” is required to ensure adequate therapeutic concentrations are maintained as the injectable medication is slowly released.^18^ Examples include risperidone, aripiprazole monohydrate, and aripiprazole lauroxil. Some risperidone LAIs do not need an oral overlap due to an early-release “double peak” property.^6^ ^,^^12^ For most LAIs, the initiation of treatment involves loading doses to achieve therapeutic concentrations more rapidly. The aripiprazole-based formulations offer optional initiation methods that avoid the need for an oral overlap.^1^ ^–^^4^ Proper adherence to these regimens is essential to bridge the interval until the LAI reaches therapeutic concentrations (Table 2).

## Postinjection monitoring

The administration of olanzapine (Relprevv) carries a <0.1% risk of post-injection delirium/sedation syndrome (PDSS), necessitating a 3-hour observation period in a registered healthcare facility with ready access to emergency response services.^5^ This monitoring allows for the detection of symptoms like excessive sedation or delirium due to rapid absorption. While PDSS is a unique risk to olanzapine (Relprevv), routine postinjection monitoring for acute adverse effects and injection site reactions is important for all LAIs.

## Management of missed doses

Maintaining a consistent schedule for LAI injections is critical, and missed appointments are a common issue. A good clinician–patient relationship is foundational to avoiding missed doses. Proactive strategies can lessen patient barriers and improve adherence. These include implementing reminder systems (phone calls, text messages), involving family or caregivers, and helping with transportation. For LAIs with long intervals between doses, reminders become even more important. Scheduling the next appointment immediately after the current one and providing a reminder card can improve follow-through. Proactive scheduling around holidays and vacations reinforces the importance of timely injections. Having a clear policy for managing missed appointments and promptly following up with patients is essential.^19^

### Rescheduling and managing delayed injections

When patients miss scheduled injections, a plan should be available to explain to the patient. Recommendations for managing missed doses vary depending on the LAI and the interval since the last injection (Table 3). Many products have a dosing window, allowing the injection to be given before or after the scheduled date. To manage a missed dose, clinicians need to know the time since the last dose, the specific product, and the dose. The appropriate action may involve administering the next injection as soon as possible, sometimes with supplemental oral antipsychotics. For certain LAIs, like paliperidone palmitate, specific re-initiation guidelines exist based on the interval since the last dose.^7^ ^–^^9^ Similarly, aripiprazole LAIs have distinct recommendations. Using pharmacy or medical records to determine the product, dose, and date of the last injection will ensure the correct catch-up protocol is used.

**Table 3.** Management of Missed Doses for Common LAIs

Medication name	Dosing window (from scheduled)	Time since last injection	Action to take
Aripiprazole Monohydrate (Abilify Maintena)	±7 d	<5 wk (2nd or 3rd dose)	Administer missed dose ASAP
		>5 wk (2nd or 3rd dose)	Administer missed dose ASAP + oral aripiprazole for 14 d
		<6 wk (≥4 consecutive doses)	Administer injection ASAP
		>6 wk (≥4 consecutive doses)	Administer next injection ASAP + oral aripiprazole for 14 d
Aripiprazole Monohydrate (Abilify Asimtufii)	±14 d	8 to 14 wk	Administer next dose ASAP
		>14 wk	Restart initiation regimen
Olanzapine Pamoate (Zyprexa Relprevv)	±28 d	≤2 mo (at steady state)	Administer next injection ASAP
		>2 mo (at steady state)	Restart loading dose regimen for 2 mo
Paliperidone Palmitate (Erzofri)	±7 d	4–6 wk	Resume regular monthly injections if stabilized on <234 mg. If stabilized on 234 mg, then: 1. 156 mg deltoid ASAP 2. 156 mg second deltoid 1 week later 3. Resume previous 234 mg regimen 1 month after second 156 mg deltoid
		>6 mo	Restart initiation regimen
Paliperidone Palmitate (Invega Sustenna)	±14 d	<6 wk (maintenance)	Administer next injection ASAP
		6 wk to 6 mo (maintenance)	1. Administer next dose in deltoid ASAP; 2. repeat same dose 1 wk later in deltoid; and 3. resume monthly
		>6 mo (maintenance)	Restart initiation regimen
Paliperidone Palmitate (Invega Trinza)	±14 d	3.5 to 4 mo	Administer next dose ASAP
		4 to 9 mo	Use special reinitiation regimen. See Table 4
Risperidone LAI (Risperdal Consta)		≤6 wk (at steady state)	Administer next injection ASAP
		>6 wk (at steady state)	Administer next injection ASAP + oral supplementation for 3 wk
Risperidone LAI (Risvan)			Administer missed dose ASAP
Risperidone LAI (Perseris)			Administer missed dose ASAP

**Table 4.** Invega Trinza 4- to 9-month Missed Dose Reinitiation Regimen

Last dose of Invega Trinza (mg)	Administer Invega Sustenna 2 doses 1 wk apart into deltoid (mg)		Administer Invega Trinza on day 36 and monthly into deltoid or gluteal (mg)
	Day 1	Day 8	
273	78	78	273
410	117	117	410
546	156	156	546
819	156	156	819

## Addressing insufficient duration of the effect

Some patients may experience symptoms returning before their next scheduled injection.^18^ ^,^^20^ This can be due to several factors, including absorption rate, metabolism, dosing, and timing. The absorption rate is a function of the product formulation, dose, injection site, and needle size. Drug interactions and genetic polymorphisms can alter the elimination rate. In most cases, the reason for early loss of effect is too low a dose, too long an injection interval, or both.

### Strategies for optimizing duration

When a patient’s duration of effect is insufficient, several strategies are available. One approach is to increase the LAI dose if it is well-tolerated. A higher dose can provide a longer period of therapeutic drug concentrations. Another strategy is to shorten the injection interval.^18^ ^,^^20^ These strategies may be “off-label” and require payer approval. For patients experiencing symptoms only toward the end of the dosing interval, supplementing with a low dose of the corresponding oral antipsychotic can serve as a bridge until a dose or interval adjustment can be made.^19^ Finally, switching to an LAI with a longer duration of action may be appropriate.

## Management of clinically significant drug interactions

Drug interactions with LAIs can lead to symptom breakthrough, increased adverse effects, or altered duration of action. These can occur through pharmacokinetic mechanisms (affecting absorption, distribution, metabolism, or excretion) or pharmacodynamic mechanisms (additive or antagonistic effects).^18^

Many antipsychotics are metabolized by cytochrome P450 (CYP) enzymes. CYP inhibitors can increase plasma concentrations, while CYP inducers can accelerate metabolism, leading to decreased concentrations and reduced efficacy.^2^ Some LAIs are also substrates of P-glycoprotein (P-gp) transporters.^21^ Clinically significant interactions include the reduction of aripiprazole and risperidone concentrations by carbamazepine and the increase in olanzapine concentrations by fluvoxamine.^18^ Pharmacodynamic interactions can also occur, such as the additive QTc prolonging effects with paliperidone.^7^ A thorough review of the patient’s complete medication history, including prescription, over-the-counter, and supplemental products, is crucial. Drug interaction databases and pharmacist consultations can aid in management (Table 5).^21^

**Table 5.** Clinically Significant Drug Interactions with Common LAIs

LAI	Interacting drug or class	Potential effect	Management recommendation
All LAIs	QT interval-prolonging drugs	Increased risk of QTc prolongation	Use with caution, monitor ECG
	CNS depressants (eg, alcohol)	Increased risk of sedation and respiratory depression	Avoid concomitant use
Aripiprazole (Abilify Asimtufii)	Strong inhibitors of CYP2D6 and CYP3A4	Increased aripiprazole exposure	Avoid use
Aripiprazole (Abilify Maintena)	Carbamazepine	Decreased aripiprazole concentrations	Consider higher dose of aripiprazole
	Strong CYP3A4 Inhibitors (eg, ketoconazole)	Increased risk of aripiprazole overexposure	Reduce aripiprazole dose
	Strong CYP3A4 Inducers (eg, rifampin)	Decreased aripiprazole concentrations	Avoid use
Olanzapine (Zyprexa Relprevv)	Fluvoxamine	Increased olanzapine concentrations	Consider lower dose of olanzapine
	Diazepam	Potentiated orthostatic hypotension	Use with caution, monitor blood pressure
Paliperidone Palmitate (Invega Sustenna)	Carbamazepine	Decreased paliperidone concentrations	Consider managing with oral paliperidone
	Strong CYP3A4/P-gp Inducers (eg, rifampin)	Decreased paliperidone exposure	Consider managing with oral paliperidone
Risperidone (Risperdal Consta)	Fluoxetine/Paroxetine	Increased risperidone concentrations	Consider lower dose of risperidone
	Carbamazepine	Decreased risperidone plasma concentration	May require higher dose of risperidone

## Addressing recreational drug use

The co-occurrence of substance use disorders is common in patients receiving LAIs and can worsen the course of illness, impact adherence, and increase relapse risk. Recreational drugs can interact with LAIs, reducing their effectiveness or exacerbating symptoms.^22^ For example, cannabis can worsen psychotic symptoms, while alcohol can amplify sedative effects. Regular, nonjudgmental assessment of substance use allows clinicians to monitor for interactions, manage symptom exacerbation, and tailor treatment strategies.^22^

## Management of common adverse effects

### Drug-induced Parkinsonism

Drug-induced Parkinsonism (DIP) is characterized by motor symptoms like tremor, rigidity, and bradykinesia.^23^ While less common with second-generation agents, it can still occur, particularly at higher doses. Management strategies include reducing the LAI dose, switching to an agent with a lower risk (eg, aripiprazole-based products), adding amantadine, or short-term use of an anticholinergic medication. Long-term anticholinergic use should be avoided due to adverse effects on cognition and an increased risk of tardive dyskinesia (Table 6).^24^

**Table 6.** Management Strategies for Common Adverse Effects of LAIs

Adverse effect	Management strategies
Drug-induced Parkinsonism (DIP)	Reduce LAI dose; switch to lower-risk antipsychotic (clozapine, quetiapine); add amantadine; consider anticholinergic (short-term)
Sedation	Reduce dose if possible; switch to less-sedating antipsychotic (aripiprazole, paliperidone)
Insomnia	Optimize timing of concomitant medications; sleep hygiene training; CBT-I; consider short-term hypnotic
Clinical evidence of prolactin elevation	Reduce LAI dose; switch to prolactin-sparing antipsychotic (aripiprazole, olanzapine); add adjunctive aripiprazole
Akathisia	Reduce LAI dose; switch to lower-risk antipsychotic; use adjunctive medication (vitamin B6, mirtazapine, or an anticholinergic) until dose reduction or switching LAI is completed
Injection site reactions	Local symptomatic treatment (ice, heat, or topical steroid); proper injection technique (site rotation, needle size); avoid massaging injection site

### Sedation

Sedation is a common side effect, particularly with agents like olanzapine. If persistent, management strategies include dose reduction or switching to a less-sedating LAI, such as aripiprazole, risperidone, or paliperidone. It is important to distinguish medication-induced sedation from the negative symptoms of schizophrenia.

### Akathisia, anxiety, or restlessness

Anxiety can be a primary symptom of schizophrenia or a side effect of antipsychotics (akathisia). If an antipsychotic was recently started or the dose increased, akathisia is likely. The best option is to reduce the LAI dose. If a rapid, short-term adjunctive medication is needed, mirtazapine, an anticholinergic, or vitamin B6 have shown efficacy, with vitamin B6 having the most favorable adverse effect profile.^25^

### Insomnia

Insomnia is a frequent issue. Management can include changing the administration time of oral sedating medications, sleep hygiene education, and cognitive behavioral therapy for insomnia (CBT-I).^26^ The short-term use of a hypnotic may be considered if other strategies are insufficient.

### Prolactin elevation

Certain LAIs, particularly risperidone and paliperidone, can elevate prolactin levels, leading to endocrine side effects.^27^ Management involves reducing the dose, switching to a prolactin-sparing agent (aripiprazole, olanzapine), or adding aripiprazole as an adjunctive medication. Regular monitoring for clinical symptoms is more beneficial than obtaining serum prolactin concentrations.

### Injection site reactions

Local reactions such as pain, redness, and swelling can occur. Proper injection technique, including using the appropriate needle size and rotating sites, can minimize these reactions. Symptomatic management typically involves local measures such as ice packs or warm compresses. These reactions usually resolve in a few days.

## Addressing patient requests to return to oral antipsychotics

Patients may request to return to oral antipsychotics for various reasons, including a mistaken belief they are “cured,” discomfort with injections, a desire for more autonomy, or concerns about adverse effects. Understanding the patient’s reasoning through a collaborative discussion is the first step. The risks and benefits of switching should be reviewed, including the increased likelihood of symptom return. The decision on when to initiate the oral medication should consider the pharmacokinetic properties of the LAI. For many second-generation LAIs, the corresponding oral medication can be started around the time of the next scheduled injection. Close monitoring for symptom recurrence is essential during the transition.

## Managing symptoms not effectively covered by an LAI

Patients on an LAI may still experience breakthrough symptoms. A systematic approach involves assessing for contributing factors like medical illness, substance use, or stressors. Optimizing nonpharmacological treatments and addressing co-occurring conditions is important. If the LAI is being administered correctly, adjusting the dose or frequency may be considered. For rapid relief, supplementation with a low dose of the corresponding immediate-release oral antipsychotic can be used.

### Integrating an oral antipsychotic for added activity

While combining antipsychotics can be viewed as inappropriate polypharmacy, judiciously adding a second medication to target specific, inadequately controlled symptoms can be a best practice. For example, patients with bipolar depression may benefit from an added oral medication, as this is not effectively covered by any current LAI (Table 7).^28^ In a patient with refractory schizophrenia on clozapine with adherence issues, an LAI can provide a safety net. A comprehensive treatment plan should also integrate nonpharmacological therapies.

**Table 7.** Medications Approved for Bipolar Disorder

Medication	Available as LAI?	For mania or mixed features	For depression
Aripiprazole	Yes	Yes	No
Asenapine	No	Yes	No
Cariprazine	No	Yes	Yes
Chlorpromazine	No	Yes	No
Iloperidone	No	No	No
Lithium	No	Yes	No
Lumateperone	No	No	Yes
Lurasidone	No	No	Yes
Olanzapine	Yes	Yes	No
Olanzapine + Fluoxetine	No	No	Yes
Paliperidone	Yes	No	No
Quetiapine	No	Yes	Yes
Risperidone	Yes	Yes	No
Valproate	No	Yes	No
Ziprasidone	No	Yes	No

## Conclusion: Optimizing LAI treatment

Successful maintenance of LAI treatment requires a comprehensive, proactive approach. This begins with a collaborative treatment plan, careful attention to preparation and administration, and effective strategies to enhance adherence. Clinicians must be prepared to manage insufficient duration of effect, potential drug interactions, and common adverse effects. Addressing patient preferences, including requests to return to oral medication, requires a non-judgmental, collaborative process. By managing these common issues, healthcare professionals can optimize LAI treatment, leading to improved clinical outcomes, enhanced quality of life, and a reduced risk of relapse for individuals living with serious mental illness.

## Data Availability

This article is based on previously published studies and does not report any new data. Therefore, no datasets were generated or analyzed.

## CNS SPECTRUMS

### CME Review Article

#### Solutions to Common Issues in the Use of Long-Acting Injectable Antipsychotics

*This CME activity is provided by HMP Education and Neuroscience Education Institute (NEI).*

### CME/CE Information

**Target audience:** This activity has been developed for the healthcare team or individual prescriber specializing in mental health. All other healthcare team members interested in psychopharmacology are welcome for advanced study.

**Learning objectives:** After completing this educational activity, you should be better able to:

- Manage common factors that can occur during long-acting injectable (LAI) antipsychotic treatment in order to optimize maintenance of treatment, including for schizophrenia and bipolar I disorder

**Accreditation:** In support of improving patient care, this activity has been planned and implemented by HMP Education and Neuroscience Education Institute (NEI). HMP Education is jointly accredited by the Accreditation Council for Continuing Medical Education (ACCME), the Accreditation Council for Pharmacy Education (ACPE), and the American Nurses Credentialing Center (ANCC), to provide continuing education for the healthcare team.

**Activity Overview:** This activity is best supported via a computer or device with current versions of the following browsers: Mozilla Firefox, Google Chrome, or Safari. A PDF reader is required for print publications. A post-test score of 70% or higher is required to receive CME/CE credit.
**Estimated Time to Complete: 1 hour.** Continuing Education credit will be available for three (3) years from the publication date of the associated article. Please visit https://nei.global/cnsspectrums2025 for additional information and to access the CE activity.

**NEI maintains a record of participation for six (6) years.*

#### Instructions for Optional Posttest and CME Credit

1. Read the article
2. Successfully complete the posttest at https://nei.global/CNS/LAI-06
3. Print your certificate

Questions? Email customerservice@neiglobal.com.

**Credit Designations:** The following are being offered for this activity:

- **Physician: ACCME *AMA PRA Category 1 Credits*™**
  - HMP Education designates this enduring material for a maximum of 1.00 *AMA PRA Category 1 Credit*™. Physicians should claim only the credit commensurate with the extent of their participation in the activity.
- **Nurse: ANCC contact hours**
  - This continuing nursing education activity awards 1.00 contact hour. Provider approved by the California Board of Registered Nursing, Provider #18006 for 1.00 contact hour.
- **Nurse Practitioner: ACCME *AMA PRA Category 1 Credit*™**
  - American Academy of Nurse Practitioners National Certification Program accepts *AMA PRA Category 1 Credits*™ from organizations accredited by the ACCME.
  - The content in this activity pertaining to pharmacology is worth 1.00 continuing education hour of pharmacotherapeutics.
- **Pharmacy: ACPE application-based contact hours**
  - This internet enduring, knowledge-based activity has been approved for a maximum of 1.00 contact hour (.10 CEU).
  - *The official record of credit will be in the CPE Monitor system. Following ACPE Policy, NEI and HMP Education must transmit your claim to CPE Monitor within 60 days from the date you complete this CPE activity and are unable to report your claimed credit after this 60-day period. Ensure your profile includes your DOB and NABP ID.*
- **Physician Associate/Assistant: AAPA Category 1 CME credits**

HMP Education has been authorized by the American Academy of PAs (AAPA) to award AAPA Category 1 CME credits for activities planned in accordance with the AAPA CME Criteria. This internet enduring activity is designated for 1.00 AAPA Category 1 credit. Approval is valid until February 11, 2028. PAs should only claim credit commensurate with the extent of their participation.

- **Psychology: APA CE credits**
  - Continuing Education (CE) credits for psychologists are provided through the co-sponsorship of the American Psychological Association (APA) Office of Continuing Education in Psychology (CEP). The APA CEP Office maintains responsibility for the content of the programs. This activity awards 1.00 CE Credit.
- **Social Work: ASWB-ACE CE credits**
  - As a Jointly Accredited Organization, HMP Education is approved to offer social work continuing education by the Association of Social Work Boards (ASWB) Approved Continuing Education (ACE) program. Organizations, not individual courses, are approved under this program. Regulatory boards are the final authority on courses accepted for continuing education credit. Social workers completing this internet enduring course receive 1.00 general continuing education credit.
- **Non-Physician Member of the Healthcare Team: Certificate of Participation**
  - HMP Education awards hours of participation (consistent with the designated number of *AMA PRA Category 1 Credit*™) to a participant who successfully completes this educational activity.

**Peer Review:** The content was peer-reviewed by an MD, LFAPA specializing in psychiatry, forensics, and addiction — to ensure the scientific accuracy and medical relevance of information presented and its independence from commercial bias. NEI and HMP Education take responsibility for the content, quality, and scientific integrity of this CME/CE activity.

**Disclosures:** All individuals in a position to influence or control content are required to disclose any relevant financial relationships. Any relevant financial relationships were mitigated prior to the activity being planned, developed, or presented.

***Faculty Author / Presenter***

**Stephen R. Saklad, Pharm.D., BCPP**

*Clinical Professor Emeritus*

Consultant/Advisor: Alkermes, BMS, Genomind, Janssen, Lundbeck, Otsuka

Speakers Bureau: BMS, Otsuka PsychU, Neurocrine, Teva

Board Member: Alkermes, BMS, Genomind

The remaining Planning Committee members, Content Editors, Peer Reviewer, and NEI planners/staff have no financial relationships to disclose. NEI and HMP Education planners and staff include Caroline O’Brien, MS, Ali Holladay, Moriah Carswell, Andrea Zimmerman, EdD, CHCP, Brielle Calleo, and Bahgwan Bahroo, MD, LFAPA.

**Disclosure of Off-Label Use:** This educational activity may include discussion of unlabeled and/or investigational uses of agents that are not currently labeled for such use by the FDA. Please consult the product prescribing information for full disclosure of labeled uses.

**Cultural Linguistic Competency and Implicit Bias:** A variety of resources addressing cultural and linguistic competencies and strategies for understanding and reducing implicit bias can be found in this handout—download me.

Accessibility Statement

For questions regarding this educational activity, or to cancel your account, please email customerservice@neiglobal.com.

**Support:** This activity is supported by an unrestricted educational grant from Alkermes, Inc., Teva Pharmaceuticals, Johnson & Johnson Innovative Medicine and Otsuka America Pharmaceutical, Inc.
